# Engineering primitive multiscale chimeric vasculature by combining human microvessels with explanted murine vessels

**DOI:** 10.1038/s41598-024-54880-6

**Published:** 2024-02-19

**Authors:** Emily A. Margolis, Lucia S. Choi, Nicole E. Friend, Andrew J. Putnam

**Affiliations:** Department of Biomedical Engineering, University of Michigan, 2204 Lurie Biomedical Eng. Bldg., 1101 Beal Ave., Ann Arbor, MI 48109 USA

**Keywords:** Regenerative medicine, Tissue engineering, Biomedical engineering, Cardiovascular biology

## Abstract

Strategies to separately manufacture arterial-scale tissue engineered vascular grafts and microvascular networks have been well-established, but efforts to bridge these two length scales to create hierarchical vasculature capable of supporting parenchymal cell functions or restoring perfusion to ischemic tissues have been limited. This work aimed to create multiscale vascular constructs by assessing the capability of macroscopic vessels isolated from mice to form functional connections to engineered capillary networks ex vivo. Vessels of venous and arterial origins from both thoracic and femoral locations were isolated from mice, and then evaluated for their abilities to sprout endothelial cells (EC) capable of inosculating with surrounding human cell-derived microvasculature within bulk fibrin hydrogels. Comparing aortae, vena cavae, and femoral vessel bundles, we identified the thoracic aorta as the rodent macrovessel that yielded the greatest degree of sprouting and interconnection to surrounding capillaries. The presence of cells undergoing vascular morphogenesis in the surrounding hydrogel attenuated EC sprouting from the macrovessel compared to sprouting into acellular hydrogels, but ultimately sprouted mouse EC interacted with human cell-derived capillary networks in the bulk, yielding chimeric vessels. We then integrated micromolded mesovessels into the constructs to engineer a primitive 3-scale vascular hierarchy comprising capillaries, mesovessels, and macrovessels. Overall, this study yielded a primitive hierarchical vasculature suitable as proof-of-concept for regenerative medicine applications and as an experimental model to better understand the spontaneous formation of host-graft vessel anastomoses.

## Introduction

Cardiovascular disease, the leading cause of death worldwide^1^, causes damage to both large-scale arteries and small-scale capillaries, but most clinical interventions (e.g., stents, grafts) focus on the macroscale vessels. In the field of tissue engineering, strategies to engineer arterial grafts^2–6^ and microvasculature^7–10^ separately have emerged, but efforts to bridge these two length scales to create multiscale vascular networks capable of supporting engineered tissues or restoring perfusion to ischemic tissues have been limited^11,12^. Such constructs could be applicable in peripheral artery disease in particular, where the arteries blocked with atherosclerotic plaque lead to further adverse effects in the downstream capillaries supplying blood and nutrients to the surrounding muscle tissue^13^. For example, pro-atherosclerotic stimuli drive a decrease in pericyte coverage of the downstream capillaries, a buildup of reactive oxygen species, and increased leukocyte recruitment. These stimuli in turn contribute to endothelial dysfunction, capillary rarefication, and decreased blood flow to the surrounding tissues. Despite interventions to reopen the atherosclerotic artery, decreased capillary density may persist, resulting in decreased blood flow and limiting the recovery of the ischemic tissue^13^.

Many studies have used aortic ring assays to study angiogenic sprouting from large, arteries^14–26^. These assays have primarily been used to study angiogenesis in a more complex, physiologic model in contrast to simplistic in vitro models that may not replicate the complexity of multiple cell types, growth factors, and extracellular matrix present in vivo. One study investigated sprouting from vessels isolated from different regions throughout the body and compared sprouting from arteries and veins^27^. Though these models do result in a multiscale construct consisting of an artery segment and sprouted capillaries, sprouting typically occurs from the lumenal end of the artery, which would diminish their capability to be sutured into circulation and used in a therapeutic application. A study employing a similar technique investigated capillary sprouting from isolated large-scale vessels to bridge artery and vein segments together^28^. In that study, an artery and vein were positioned on either side of a micropatterned hydrogel and angiogenic factors were employed to induce sprouting between the two segments and ultimately connect them. While this approach would be more translatable due to a free, suturable end on both sides of this multiscale construct, it would function more like a bridge across the arterial circulation as the small-scale capillaries connecting the two large vessels did not branch to vascularize the surrounding tissue.

The work presented here aimed to create hierarchical vascularized tissue constructs spanning multiple length scales by evaluating the ability of vessels isolated from mice to sprout and form functional connections to engineered human capillary networks ex vivo. We demonstrated sprouting from the abluminal edges of quiescent macroscopic murine vessels, and showed that human cell-derived microvasculature in a surrounding hydrogel can inosculate with these large vessel-derived sprouts. The experimental model developed here also provides a platform to better understand the mechanisms of inosculation that lead to the spontaneous formation of host-graft anastomoses, which remain poorly understood.

## Results

### Large vessels sprout into acellular fibrin hydrogels

**Figure 1:**
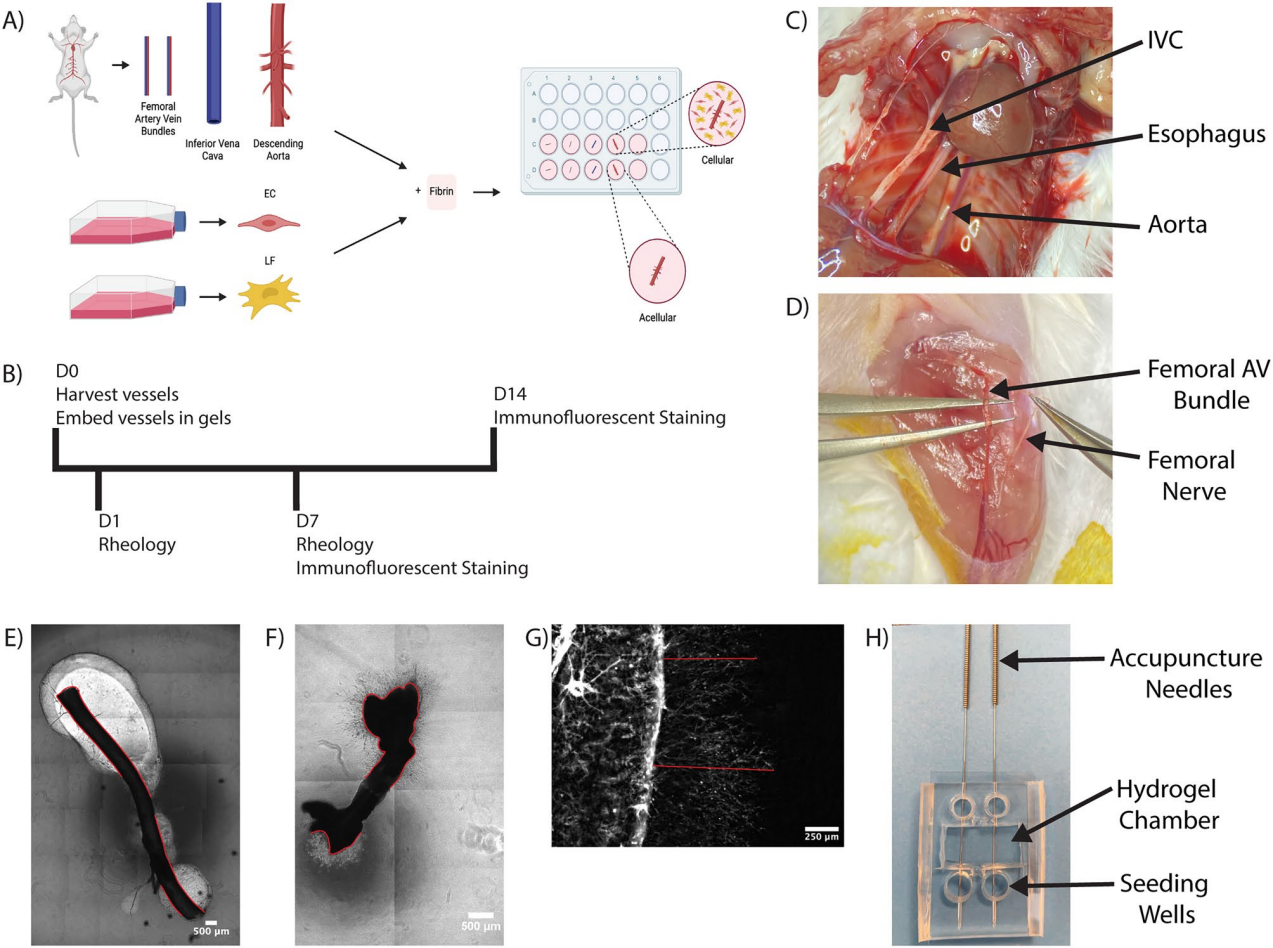
Methodology for the fabrication and analysis of hierarchical vascular networks containing explanted murine macrovessels. (**A**) Schematic diagram of cell and vessel harvest procedures and hydrogel fabrication approach. Schematic created using BioRender.com. (**B**) Experimental timeline. (**C**) Surgical view of thoracic cavity showing the two explanted vessels and the esophagus which was avoided. (**D**) Surgical view of the left femoral muscle showing the AV bundle and the femoral nerve which was avoided. (E–G) Methods for analysis of explants. (**E**) Degradation analysis—red lines indicate degraded perimeter. (**F**) Percent sprouted analysis—red lines indicate sprouted perimeter (**G**) Sprout distance analysis—red lines indicate sample traces of cell sprouting. (**H**) Color image of the PDMS mold for fabricating 3-scale vascular hierarchies bonded to glass with needles inserted.

**Figure 2:**
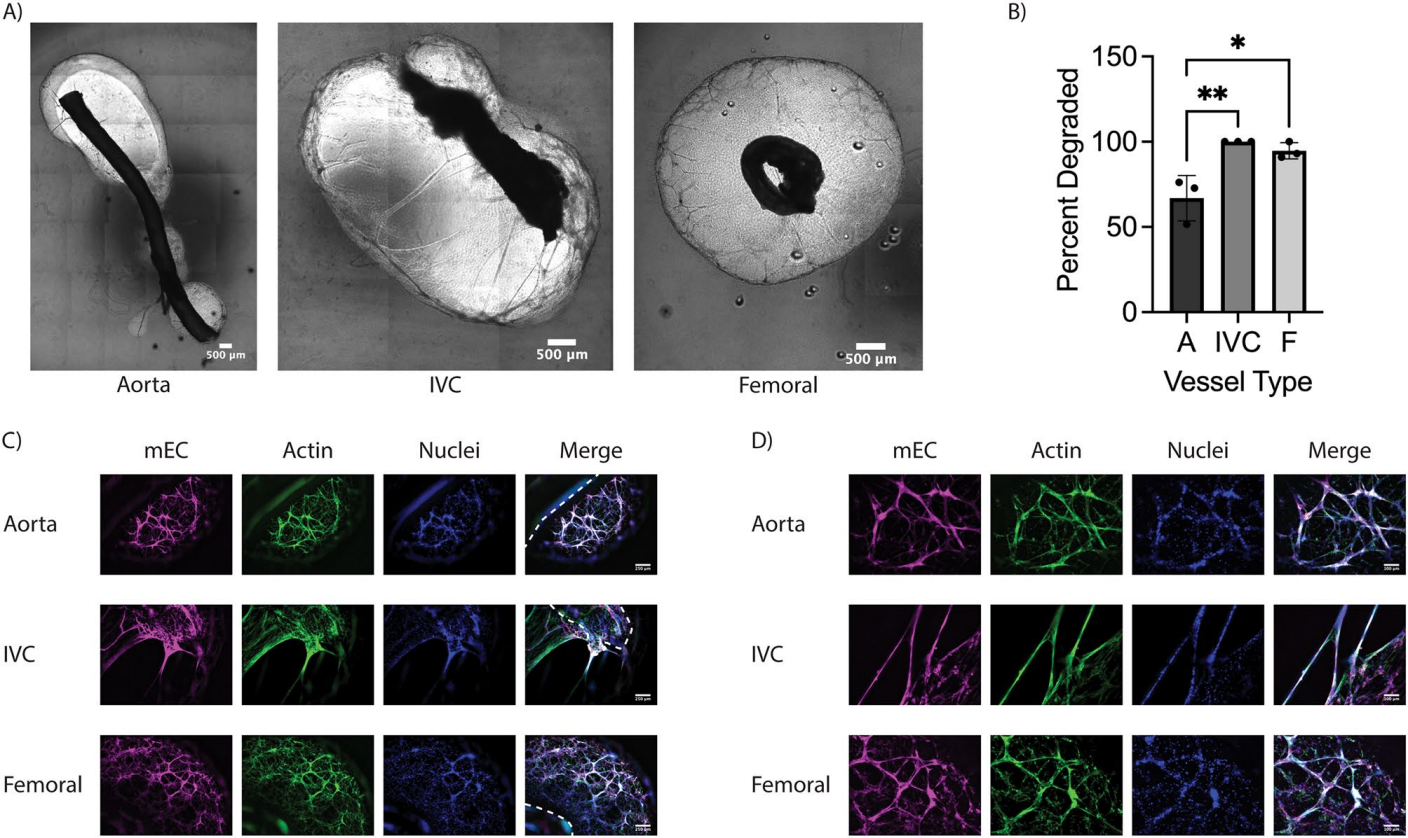
Explanted macrovessels embedded in acellular fibrin hydrogels cause significant fibrinolysis. (**A**) Macroscopic phase contrast images of the entire explant and surrounding degraded fibrin hydrogel. (**B**) Quantification of explant perimeter with degraded fibrin. (n = 3; *p < 0.05, **p < 0.01) (**C**) Representative fluorescent images of sprouting from the explanted vessel edge into degraded fibrin regions. In some images, the explanted vessel edges are demarcated by dashed lines. (Magenta: mouse EC (GSL), Green: F-actin (phalloidin), Blue: Nuclei (DAPI). Scale bars = 250 µm). (**D**) Magnified view of vessel-like structures. (Scale bars = 100 µm).

We examined sprouting from murine aortae, vena cavae, and femoral arteriovenous (AV) bundles embedded in fibrin hydrogels (Fig. 1). Significant sprouting was observed from all three types of vessels into acellular fibrin hydrogels, with both stromal cells (SC) and EC emanating from all three vessel types to invade the fibrin. Mouse EC labeled with a mouse-specific lectin (GSL+) were localized in close proximity to non-endothelial (GSL−) cells (Fig. 2C,D), suggestive of a perivascular association of mouse SC. Explants embedded in acellular hydrogels sprouted in a collective, multicellular morphology (Fig. 2C,D), but significantly degraded the fibrin surrounding the vessel (Fig. 2A). There was significantly less fibrinolysis around aortae compared to vena cavae and AV bundles (Fig. 2B). Sprouting primarily occurred in the degraded regions of the hydrogel, resulting in cord-like endothelial structures that appeared to stretch across areas devoid of matrix. The nuclear staining showed clusters of cells rather than a vessel-like arrangement with a hollow lumen between nuclei (Fig. 2D, Nuclei). These constructs were not further analyzed for sprouting metrics as the large degree of fibrinolysis would limit their utility as transplantable engineered vascularized tissue constructs.

**Figure 3:**
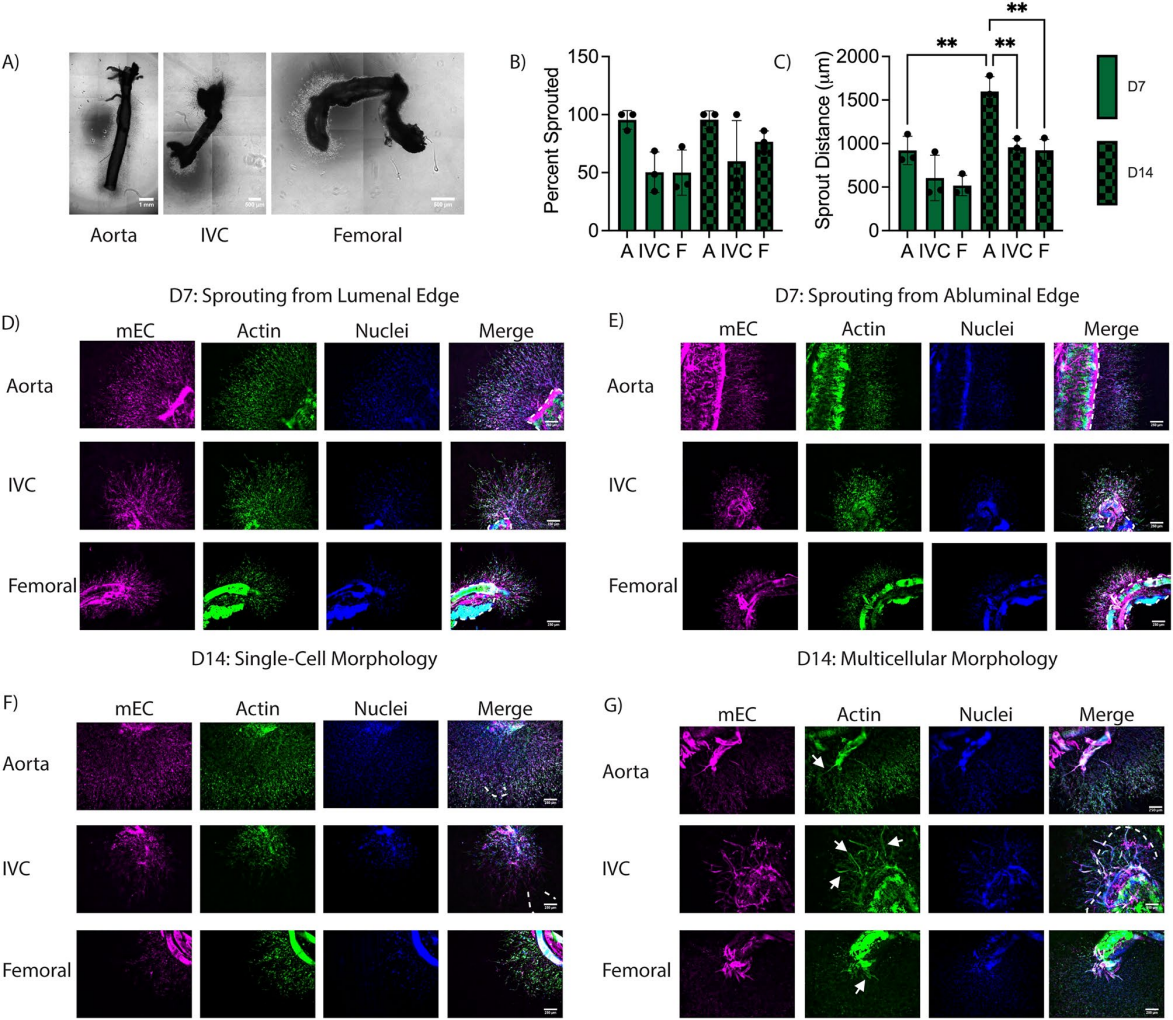
Explanted macrovessels embedded in acellular fibrin hydrogels in the presence of aprotinin yield significant sprouting from the abluminal and lumenal edges. (**A**) Macroscopic phase contrast images of the entire explant and surrounding fibrin hydrogel. (**B**) Quantification of explant perimeter with sprouted cells (n = 3). (**C**) Quantification of mEC sprout distance from explant edge. (n = 3; **p < 0.01) (**D**) Representative fluorescent images of sprouting from the luminal explant edge (the cut end) at D7. (**E**) Representative fluorescent images of sprouting from the abluminal explant edge (along the length of the vessel) at D7. (**F**) Representative fluorescent images of sprouting from the explant edge at D14 with the same morphology as at D7. (**G**) Representative fluorescent images of sprouting from the explant edge at D14 with a distinct multicellular morphology. White arrows denote multicellular sprouts. In some images, the explanted vessel edges are demarcated by dashed lines. (Magenta: mouse EC (GSL), Green: F-actin (phalloidin), Blue: Nuclei (DAPI). Scale bars = 250 µm).

To mitigate the significant fibrinolysis, aprotinin, a protease inhibitor, was added to the culture medium. In the presence of aprotinin, explants did not degrade the surrounding fibrin (Fig. 3A), and sprouts were morphologically different in the surrounding hydrogel. In contrast to the multicellular clusters observed without aprotinin, explants cultured in fibrin gels in the presence of aprotinin sprouted as single cells of both endothelial and stromal origins (Fig. 3D,E). All vessels sprouted from the cut, lumenal edge (Fig. 3D) in addition to the abluminal edge (Fig. 3E). Though all vessels sprouted, aortae sprouted from a greater percentage of the vessel perimeter (Fig. 3B) and showed a trend of increased sprout distance from the vessel edge compared to vena cavae and femoral AV bundles (Fig. 3C). Similar to the results in Fig. 2, mouse EC labeled with a mouse-specific lectin (GSL+) were localized in close proximity to non-endothelial (GSL−) cells (Fig. 3D–G).

When cultured for an additional week, explants continued to sprout further into the hydrogel, resulting in sprout distances from aortae that were significantly greater than those from the other explanted vessel types (Fig. 3C). Femoral bundles and vena cavae displayed increased sprout distance, though the differences between days 7 and 14 for these vessels were not statistically significant. Additionally, sprout distances from aortae at day 14 were significantly greater than the values at day 7. The dominant sprouting morphology at day 14 remained single cells (Fig. 3F), but in some instances, there was evidence of multicellular cluster sprouting predominantly from the cut edge (Fig. 3G, white arrows). This morphology was observed more from vena cavae and femoral AV bundles than aortae, and was only observed in approximately two thirds of the vessels of each type analyzed. When this morphology was observed from aortae, it typically occurred from the ends of intercostal branches (Fig. 3G, Aorta), rather than the main trunk of the aorta. Additionally, the multicellular sprouts from vena cavae and aortae tended to be thinner than those from femoral bundles.

### Sprouting persists into cell-laden fibrin hydrogels to create chimeric microvasculature

**Figure 4:**
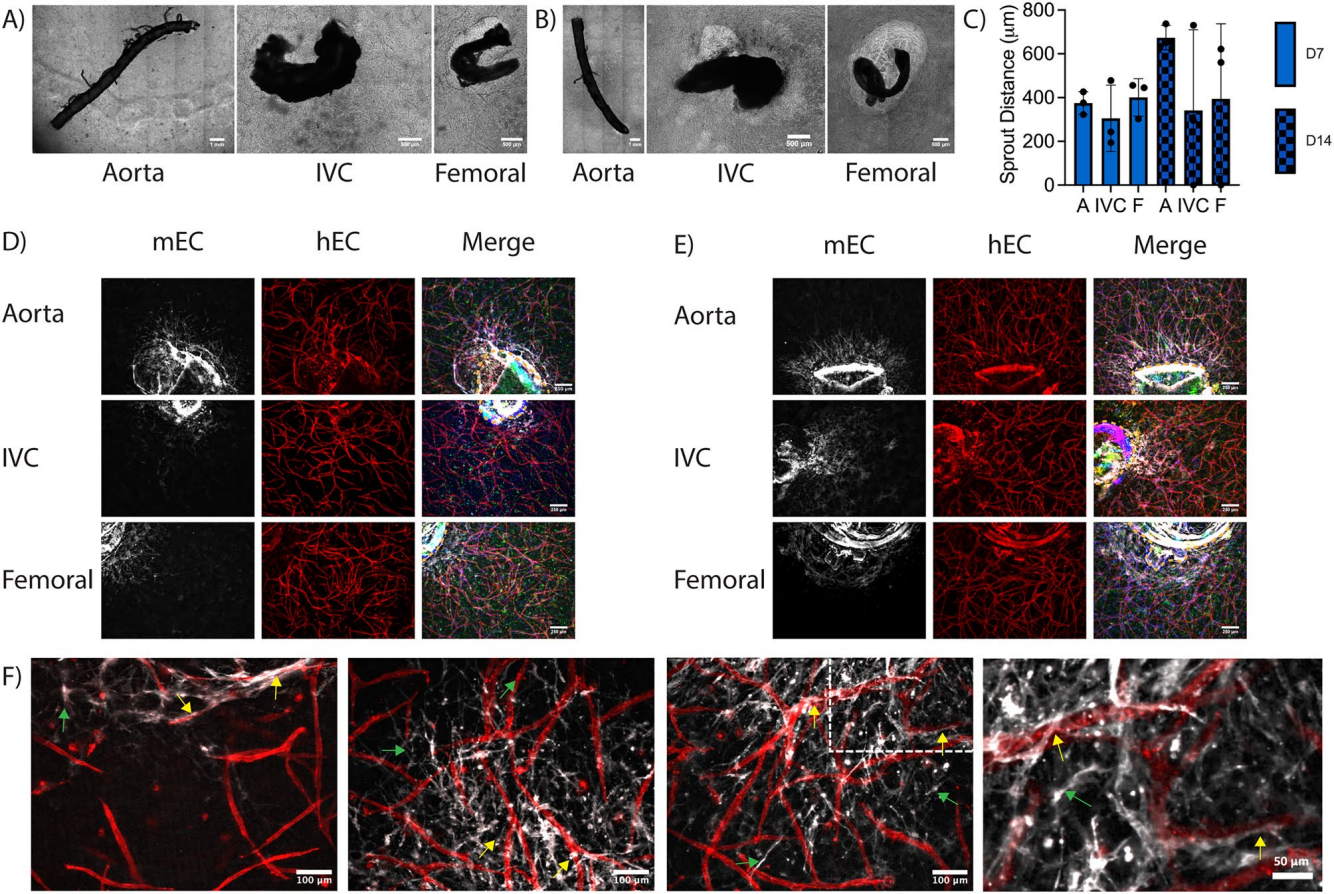
Formation of chimeric vascular structures. (A, B) Macroscopic phase contrast images of the entire explant and surrounding cell-laden fibrin at (**A**) D7 and (**B**) D14. (**C**) Quantification of mEC sprout distance from the explant edge into the hydrogel. (D, E) Representative fluorescent images of mEC sprouting from explants at (**D**) D7 and (**E**) D14. In some images, the explanted vessel edges are demarcated by dashed lines. (Red: human EC (UEA), White: mouse EC (GSL); Green: F-actin (phalloidin), Blue: Nuclei (DAPI). Scale bars = 250 µm.) (**F**) Representative magnified fluorescent images of mEC-hEC interactions and morphologies. The fourth panel represents a magnified version of the boxed region in the third panel. (Yellow arrows—mEC interacting with hEC. Green arrows—mEC sprouted into the hydrogel not interacting with hEC. Scale bars = 100 µm).

We next examined how these EC sprouts from explants interact with human EC and SC undergoing vasculogenic self-assembly in the surrounding fibrin matrix without the inclusion of aprotinin. We’ve previously shown that co-embedding HUVEC and LF in 3D fibrin gels results in a vasculogenic self-assembly process characterized by the formation of lumenized structures, the presence of a basement membrane, and differentiation of the LF into perivascular support cells that express αSMA, NG2, and PDGFRβ^29,30^. Mouse EC from explanted vessels continued to sprout into cell-laden fibrin hydrogels. The presence of human cells in the bulk hydrogel attenuated explant-induced fibrinolysis at day 7 (Fig. 4A), but there was some evidence of fibrinolysis at day 14 (Fig. 4B). This degradation was inconsistent across explant types, with femoral and vena cavae explants exhibiting more degradation that aortae, consistent with the degradation observed in acellular hydrogels (Fig. 2). Species-specific markers were used to label the different populations of EC, and showed that the human EC in the bulk hydrogel self-assembled into capillaries (Fig. 4D,E, hEC) over the course of culture coincident with sprouting of the mouse EC into the hydrogel (Fig. 4D,E, mEC). In regions that did not degrade, the EC sprouting morphology was comparable to that of the explants in acellular hydrogels cultured in the presence of aprotinin (Fig. 4D compared to Fig. 3D,E). In regions that did degrade, the sprouting morphology was comparable to the acellular sprout morphology (Fig. 4E femoral compared to Fig. 2C,D). Sprouting occurred from both lumenal and abluminal edges, though the number of sprouted regions was attenuated in comparison to the acellular samples cultured in the presence of aprotinin.

**Figure 5:**
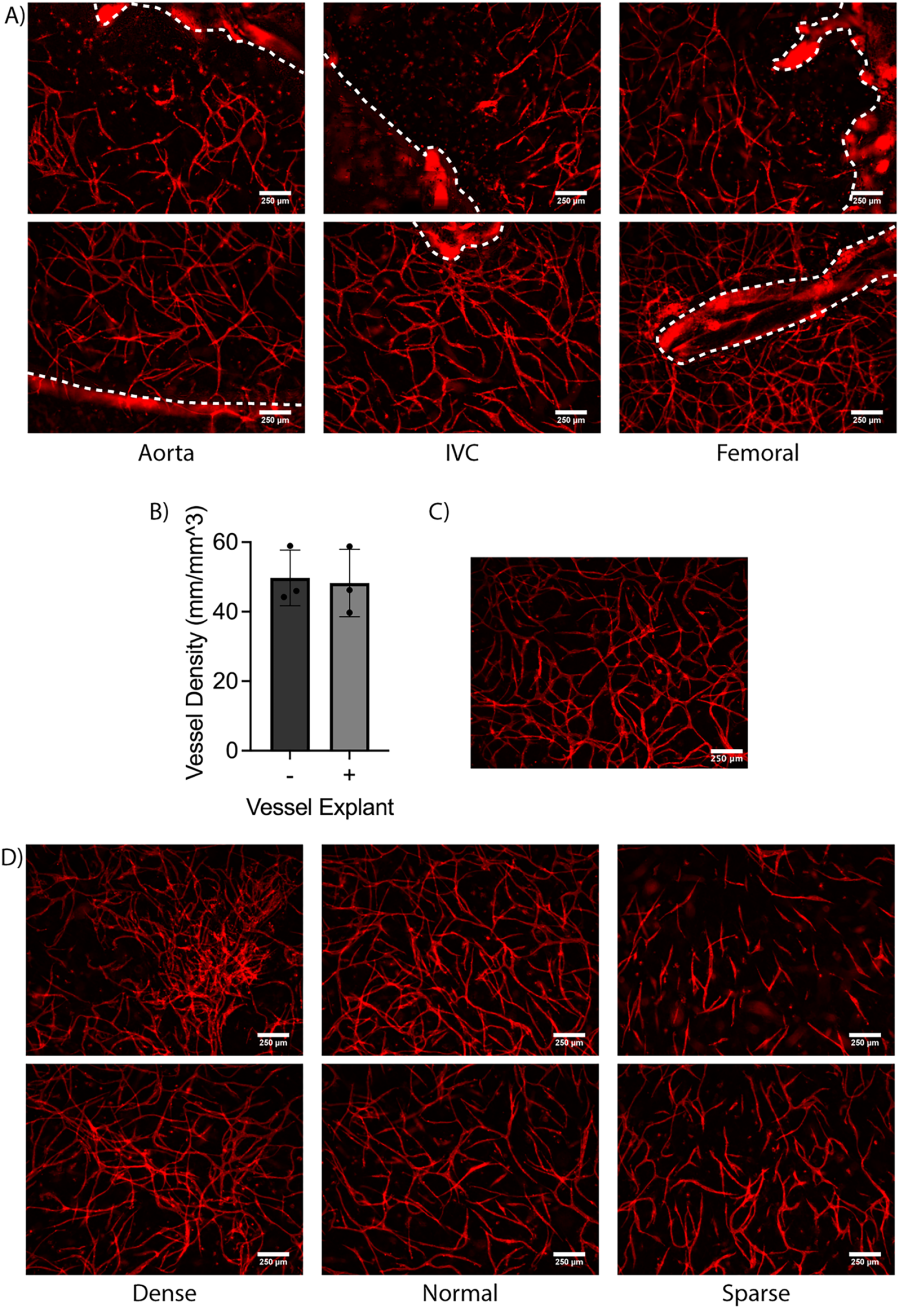
Macrovessel explants induce changes to capillary morphology but not overall microvascular density in cell-laden fibrin hydrogels. (**A**) Representative images of human self-assembled capillaries formed around explants. Dashed lines demarcate explanted macrovessel edge. (Red: human EC (UEA). Scale bars = 250 µm). (**B**) Quantification of microvascular density in cell-laden fibrin hydrogels with or without explants. N = 3 vessels from 3 separate mice per vessel type. N = 3 independent replicates examined. Vessel explant types merged for each replicate. (**C**) Representative image of capillaries in a hydrogel without an explant. (**D**) Representative images of three different capillary morphologies. (Red: human EC (UEA). Scale bars = 250 µm).

At higher magnification, two different migration morphologies were observed. In some cases, mouse EC sprouted into the gel without interacting with human EC (Fig. 4F, green arrows). These mEC in the hydrogel exhibited a spindle-like morphology, somewhat akin to that of fibroblasts. In other cases, mouse EC sprouted towards and began interacting with and wrapping around the human EC-derived capillary-like structures (Fig. 4F, yellow arrows). These mEC appeared elongated along the hEC-derived vessels, adopting a perivascular morphology in which the mEC appeared to wrap around and ensheath the hEC-derived vessels. Similarly, the presence of mouse large vessel explants within the hydrogels also induced morphological changes to the human vasculature. At regions close to the explant, all three explant types showed evidence of human vasculature directly next to the explant (Fig. 5A, bottom); however, some regions contained single cells that were not self-assembled into microvasculature immediately adjacent to the explanted mouse vessels (Fig. 5A, top). In the bulk of the hydrogel, at distances > 1 mm from the explant edge, the density of human vasculature was comparable to gels without explants (Fig. 5B,C). However, explants did induce some morphological differences in vascular assembly throughout the bulk hydrogel. Most regions contained a normal vascular density (Fig. 5D, middle) comparable to gels without an explant (Fig. 5C), but there was more spatial heterogeneity in vascular densities observed in constructs containing mouse explants with some regions containing more dense microvasculature (Fig. 5D, left) and other regions with more sparse microvasculature (Fig. 5D, right).

**Figure 6:**
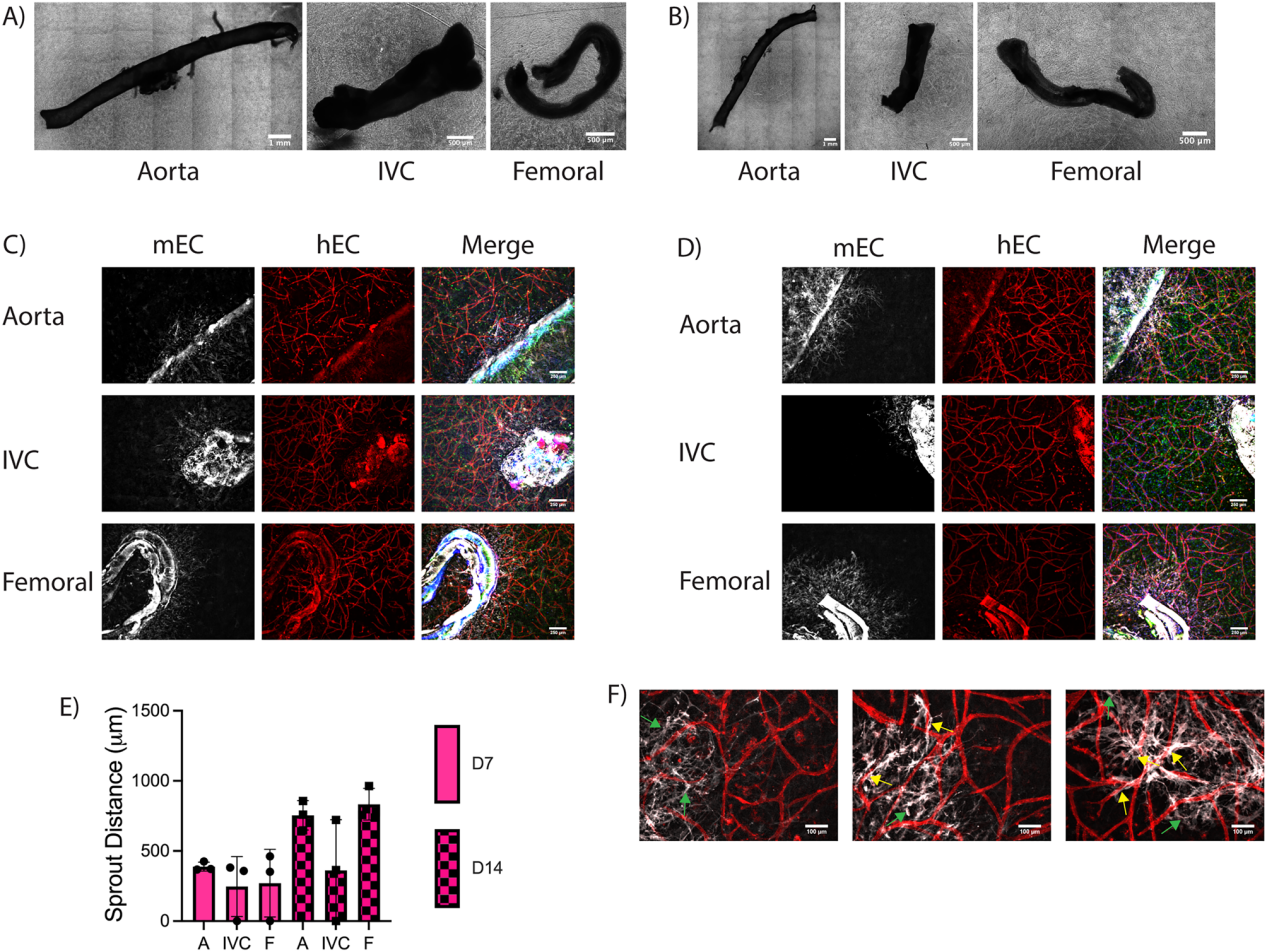
Formation of chimeric vascular structures in cell-laden hydrogels with aprotinin supplement. (A, B) Macroscopic phase contrast images of the entire explanted macrovessels and surrounding cell-laden fibrin at (**A**) D7 and (**B**) D14. (C, D) Representative fluorescent images of mEC sprouting from explants at (**C**) D7 and (**D**) D14. (Red: human EC (UEA), White: mouse EC (GSL); Green: F-actin (phalloidin), Blue: Nuclei (DAPI). Scale bars = 250 µm). (**E**) Quantification of mEC sprout distance into the hydrogel. (**F**) Representative magnified fluorescent images of mEC-hEC interactions and morphologies. (Yellow arrows—mEC interacting with hEC. Green arrows—mEC sprouted into the hydrogel not interacting with hEC. Scale bars = 100 µm).

Comparable to the effect in acellular gels, the inclusion of aprotinin attenuated explant-induced fibrinolysis in cell-laden hydrogels (Fig. 6A,B). When aprotinin was incorporated into the media for cell-laden hydrogels, the sprout morphologies remained unchanged compared to those without aprotinin; mEC still sprouted as single cells (Fig. 6C,D) to similar distances (Fig. 6E). In cultures containing aprotinin, chimeric vasculature was also observed with some mEC sprouting towards and wrapping around hEC-derived microvessels (Fig. 6F, yellow arrows).

### Human cells attenuate sprouting from murine explants

**Figure 7:**
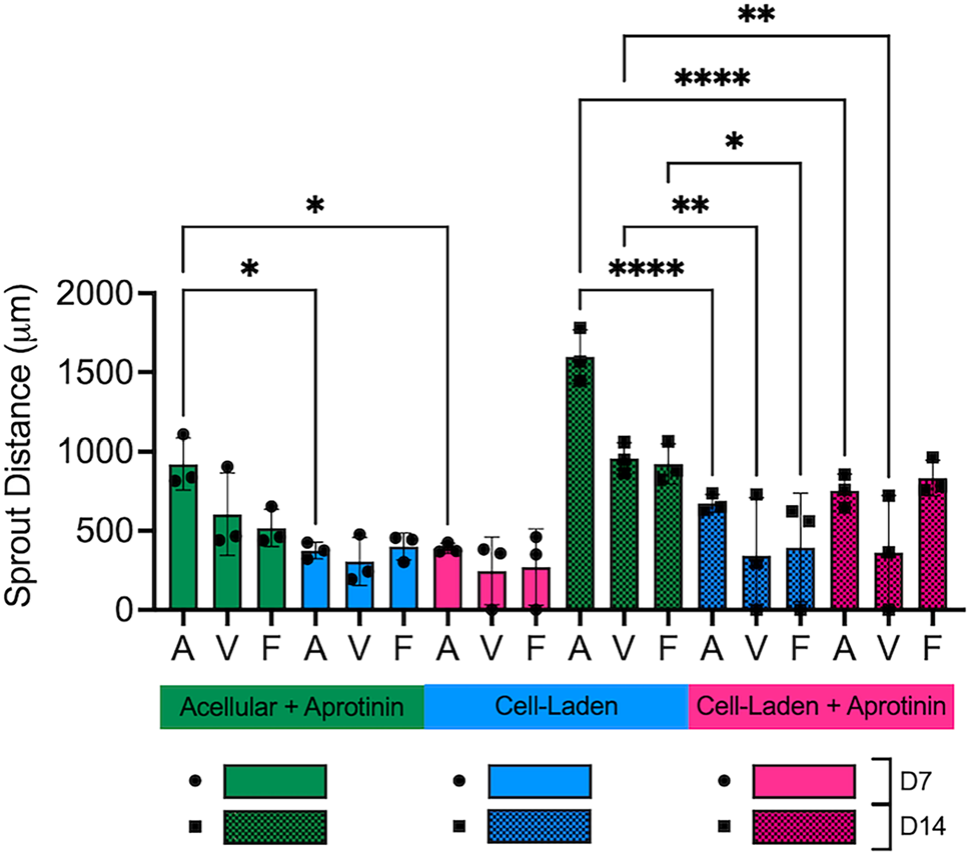
The presence of human cells in the bulk hydrogels diminishes mEC sprout distance. Quantification of mEC sprout distance into fibrin hydrogels of different compositions. Quantitative data from figures 4.3C, 4.4C, and 4.6E combined here for clarity. (n = 3; *p < 0.05, **p < 0.01, ****p < 0.0001).

Quantification of sprout distance for all three conditions (Acellular + Aprotinin, Cell-laden, and Cell-laden + Aprotinin) demonstrated that the presence of human cells in the bulk hydrogel attenuated sprouting from the large mouse vessels over time relative to sprouting into acellular hydrogels (Fig. 7). At day 7, aortae sprouted significantly less in both cell-laden conditions compared to acellular hydrogels with the aprotinin supplement. At day 14, all three vessel types and both thoracic vessels displayed significantly attenuated sprouting in cell-laden hydrogels and cell-laden hydrogels with the aprotinin supplement, respectively, compared to acellular hydrogels with the aprotinin supplement. Further, the presence of aprotinin did not affect sprout distance in cell-laden hydrogels with no significant differences observed between these conditions.

### Sprout distance is correlated with changes in bulk stiffness

**Figure 8:**
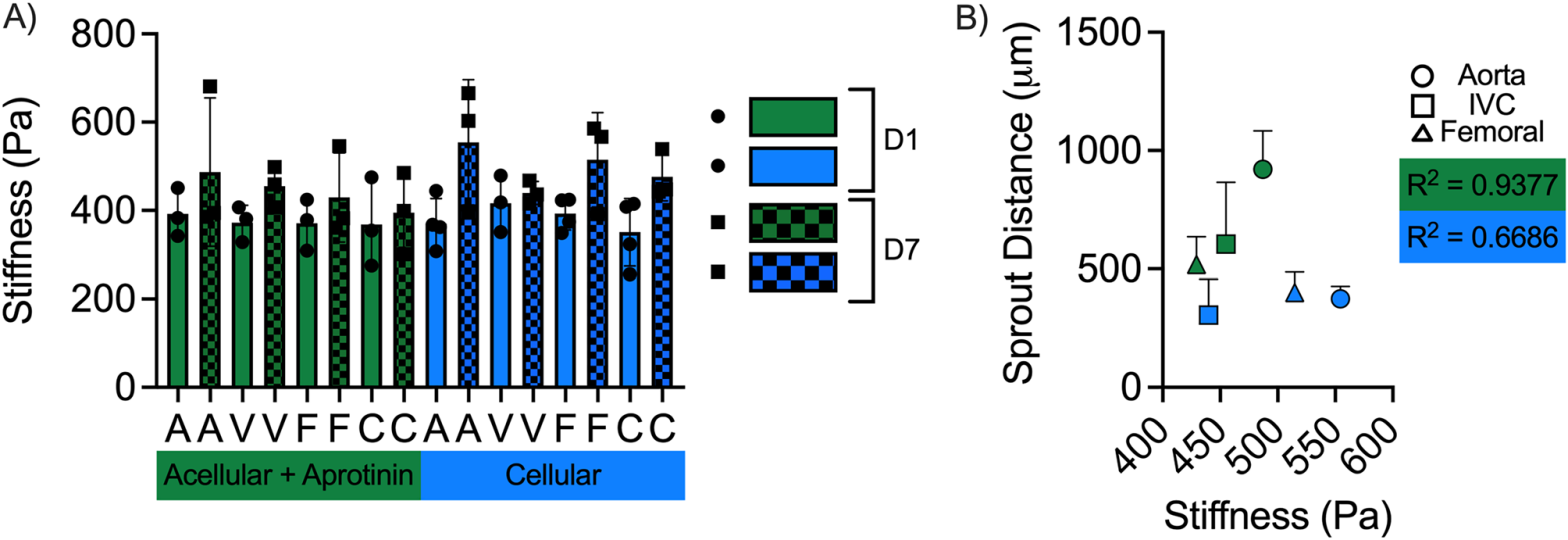
Sprout distance and hydrogel stiffness are correlated in some conditions. (**A**) Quantification of bulk hydrogel stiffness (G’) via rheology. (**B**) Correlation between sprout distance and hydrogel stiffness at day 7. Sprouting of mouse EC into acellular fibrin gels in the presence of aprotinin is strongly correlated with bulk stiffness. (Green data: acellular + aprotinin samples; Blue data: cell-laden samples).

We examined hydrogel mechanical properties via parallel plate rheometry to determine if explant sprouting induced changes in hydrogel stiffness. The presence of a large vessel explant within the hydrogel did not cause any significant differences in the shear elastic modulus G’, a measure of the bulk stiffness, on day 1 (Fig. 8A). Sprouting into the hydrogels also did not result in any significant changes in hydrogel stiffness in either acellular hydrogels with the aprotinin supplement or cell-laden hydrogels after 7 days of culture (Fig. 8A). Though not significant, most groups did show a slight increase in stiffness from day 1 to day 7. We then evaluated if there was a linear correlation between hydrogel stiffness and sprout distance after 7 days in culture. In the acellular + aprotinin conditions in which sprout distances were significantly larger from aortae versus venae cavae and femoral vessels (Fig. 3C), there was a strong correlation between sprout distance and hydrogel stiffness, with an R^2^ value of 0.9377 (Fig. 8B). In the cell-laden hydrogels where sprout distances were statistically equal amongst the different vessel types (Fig. 4C), sprout distance and hydrogel stiffness were not well correlated as evidenced by an R^2^ value of 0.6686 (Fig. 8B).

### Proof-of-concept: creating primitive three-scale vascular hierarchies

**Figure 9:**
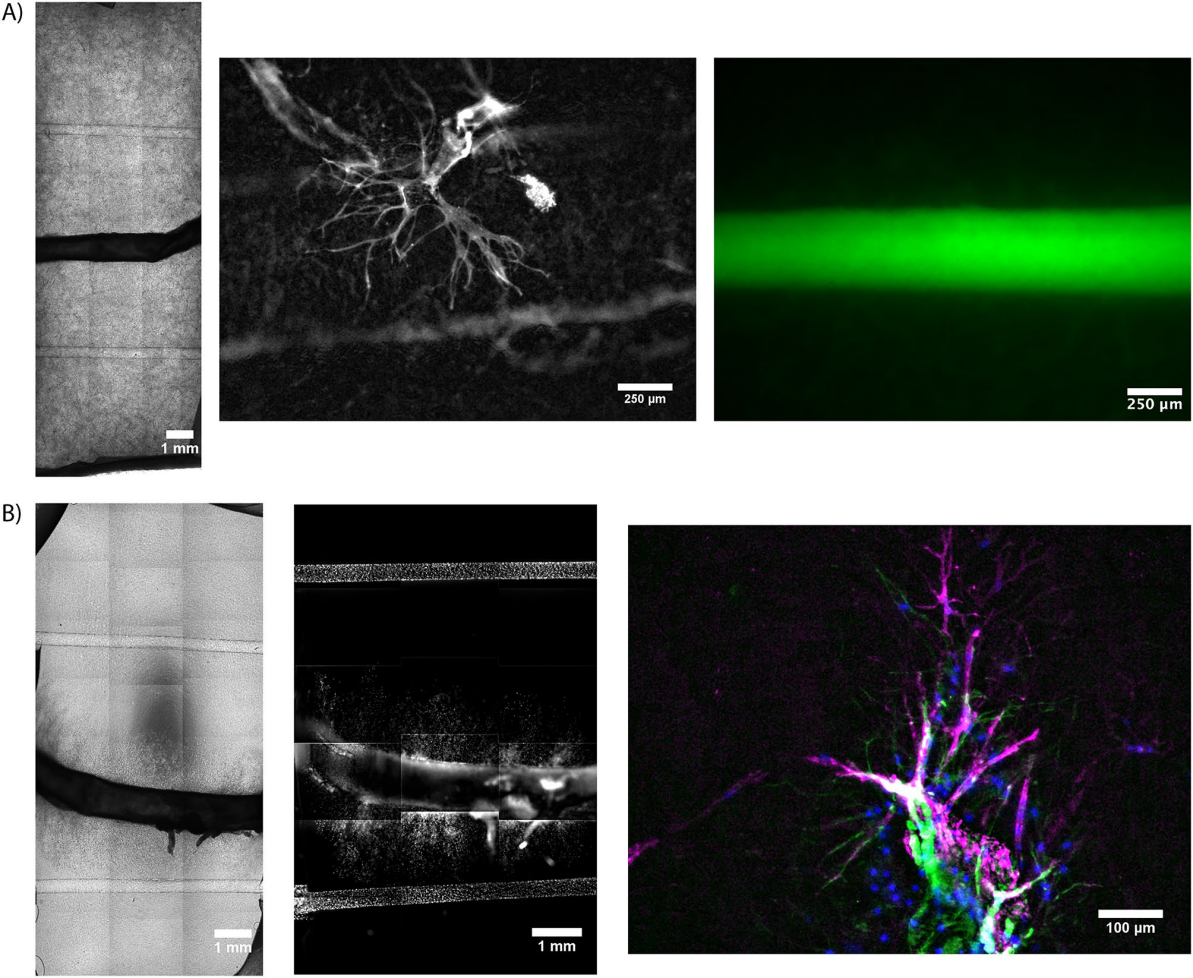
Engineered primitive multiscale vascular hierarchies with murine aortas as a macrovessel. (**A**) Aorta embedded in cell-laden fibrin hydrogels. Whole sample phase contrast scan (left), mEC vessel-like sprout morphology (middle panel; white: mouse EC (GSL)), dextran perfusion through mesovessel (right panel; green: FITC dextran). (**B**) Aorta embedded in acellular fibrin hydrogel supplemented with aprotinin. Whole sample phase contrast scan (left) and nuclei scan (middle panel; white: nuclei (DAPI). High magnification view of vessel-like sprouts (right panel; *Magenta: mouse EC (GSL), Green: F-actin (phalloidin), Blue: Nuclei (DAPI)*).

One major drawback of most current vascularized tissue constructs is the lack of a multiscale vascular hierarchy within the tissue construct. As an initial step towards this goal, we sought to incorporate mesovessels within our constructs to fabricate a more complete hierarchy containing all three vascular length scales (Fig. 9). Combining the mouse aorta as a model large and suturable vessel into a hydrogel with self-assembled capillaries and two mesovessels, we created a complete vascular hierarchy containing a microvascular network, mesovessels, and a macrovessel. Both cell-laden hydrogels (Fig. 9A) and acellular hydrogels supplemented with aprotinin were used to create these hierarchies (Fig. 9B). The same sprouting morphologies as previously shown in day 7 cell-laden constructs (Fig. 4) were observed, along with a vessel-like phenotype not previously observed in day 7 cell-laden constructs (Fig. 9A, middle). The mesovessels were perfusable (Fig. 9A, right), but functional inosculation between the meso- and microvasculature, as previously shown by our group^30^, was not observed here. The acellular + aprotinin sample was cultured for 21 days to allow sprouted cells to reach the mesovessel and integrate (Fig. 9B, left and middle). We observed the same multicellular phenotype shown previously at day 14 from an intercostal branch (Fig. 9B, right).

## Discussion

The engineering of hierarchical vasculature is a critical step to advance engineered tissues and organs closer to clinical translation. Although effective strategies to engineer vascular hierarchies remain limited, combinations of top-down (e.g., TEVGs) and bottom-up (e.g., vascular self-assembly) approaches are beginning to show promise^31^. However, the integration of vessels across length-scales, especially in the context of vascularized engineered tissue grafts, remains incompletely understood. In this study, we evaluated the ability of macroscale rodent vessels (murine arteries and veins from both thoracic and femoral origins) to interact with human cell-derived microvasculature ex vivo as a step towards engineering a multiscale vascular hierarchy and achieving a better understanding of the inosculation process. Despite the dogmatic view that large vessels are incapable of undergoing angiogenic sprouting due to their thick medial layers of smooth muscle, or only do so from cut or damaged ends as observed in aortic ring assays, we observed sprouting of endothelial cells from both the abluminal edges and the luminal cut edges of murine aortae, vena cavae, and femoral AV bundles into both acellular and cell-laden fibrin hydrogels. Furthermore, we observed evidence of inosculation between these nascent sprouts with self-assembled human microvasculature to yield a primitive, chimeric, hierarchically vascularized tissue construct.

Sprouting from the mouse macrovessels into acellular fibrin gels was accompanied by significant explant-induced fibrinolysis. Despite notable voids at the interface between the explanted vessels and the fibrin gels, we observed the presence of multicellular web-like structures containing mouse EC that emanated from the macroscale vessels and bridged the void to interact with the fibrin gel. In the presence of aprotinin, a broad-spectrum inhibitor of serine proteases (especially plasmin), these large voids were attenuated. Sprouting of mouse cells (including EC) into the fibrin still occurred, but mostly as discrete cells. Despite differences in EC sprout morphologies +/− aprotinin, sprouting morphologies were qualitatively consistent across the different vessel types examined. Similar sprouting phenomena in the presence of aprotinin have been previously reported in aortic ring assays^32^, reinforcing known roles for the matrix metalloproteinases (MMPs) in the initiation of microvessel sprouts, and in their regression at later timepoints^33,34^. Although plasmin-mediated thrombolysis is an important aspect of fibrin clot breakdown in the vasculature, a prior study showed that angiogenic sprouting from aortic rings from plasminogen knock-out mice was indistinguishable from those isolated from control mice, suggesting that angiogenic sprouting into the interstitial matrix can still occur in the absence of plasmin-mediated fibrinolysis and instead requires the activity of the membrane-anchored MT1-MMP^35^. Similarly, earlier work from Hiraoka, et al. investigating angiogenesis from aortic rings in fibrin gels reported that neither plasminogen nor plasminogen activator were required for neovessel formation, but rather MMPs were critical regulators of vessel sprout formation in fibrin hydrogels^36^. Prior work from our own group has also established that microvascular sprouting in fibrin occurs in the presence of aprotinin, but can be nearly completely abrogated when both plasmin- and MMP-mediated fibrinolysis are blocked^37^. Although the prior studies point to an MMP-dependent mechanism, our observation that mouse EC from aortae, vena cavae, and femoral AV bundles sprout into fibrin often via a different morphology in the presence of aprotinin leaves open the possibility of cross-talk between MMP- and plasmin-mediated degradative mechanisms^38,39^.

We also observed quantitative differences in the sprouting percentage (i.e., percentage of a vessel perimeter exhibiting sprouting) and sprout distance from aortae, vena cavae, and femoral AV bundles into acellular fibrin hydrogels in the presence of aprotinin, with both metrics showing that sprouting was greatest from aortae. There are several possible explanations for these quantitative differences, including the fact that these different vessels have different starting compositions in terms of both cells (endothelial, smooth muscle, fibroblast, and immune cells) and extracellular matrix which may influence their ability to sprout. While muscular arteries have a thicker vessel wall with greater numbers of cells compared to thinner-walled veins, they also typically have their own network of capillaries called the vasa vasorum within the outermost layers (i.e., adventitia) of the vascular wall. The sprouting observed from the aortae into the surrounding fibrin hydrogels may have originated from this vasa vasorum. The sprouting potential may have also been affected by the presence of tissue layers around the vessels. During manual dissection, removal of the connective tissue and/or muscle tissue around the explanted vessels required significant mechanical manipulation. Being a strong muscular artery, the aortae could withstand the physical forces involved in the manual dissection without sustaining too much damage. In contrast, vena cavae have thin, non-muscular walls that could be easily ripped due to the forces associated with manual dissection and removal of connective tissue. Because of their relative fragility, these vessels were not cleaned as thoroughly as aortae, potentially leaving residual adventitia and/or connective tissue on the outside of the vessel wall that may have acted as a barrier to sprouting. Similar results were reported by Vaghela, et al. comparing the ex vivo ring assay with vessels of different origins^27^.

The presence of human cells in the fibrin also attenuated implanted-induced fibrinolysis, enabling formation of a mechanically stable construct. This may be due to the expression of MMP inhibitors (TIMPs) and/or plasminogen inhibitors by these human cells. For example, endothelial expression of plasminogen activator inhibitor (PAI)-1 has been shown to slow plasmin-mediated fibrinolysis^40^. Mouse EC sprouted into fibrin gels containing human cell-derived microvascular networks, but the magnitude of mouse EC sprouting was less than in acellular gels supplemented with aprotinin (comparing Figs. 3C and 4C). Differences in nutrient and/or pro-angiogenic growth factor gradients may potentially explain these observations. In acellular hydrogels, mouse cells are inclined to directionally sprout from the explanted vessels into the hydrogel towards higher concentrations of these cues, as evidenced by increased EC sprouting between day 7 and day 14. Similar gradient-induced angiogenic sprouting has been described previously wherein cells migrate from one vessel to another via a source of nutrients and growth factors^41,42^. In contrast, in cell-laden hydrogels, human cells in the bulk consume nutrients and growth factors, so any gradients that exist would presumably be much shallower and perhaps less capable of driving murine EC sprouting into the cell-laden hydrogels. This gradient steepness argument is also consistent with a prior report that endothelial cells migrate maximally in response to sharp gradients of VEGF and FGF-2, rather than in response to the peak concentration of these pro-angiogenic factors^43^.

We also examined changes in the bulk rheological properties of the fibrin hydrogels with and without macrovessel explants. Our analysis identified no significant differences in hydrogel stiffness between day 1 (pre-sprouting) and day 7 hydrogels in both acellular samples with the aprotinin supplement and cell-laden samples. However, when parsed based on the identity of the vessel explant, our analysis yielded a strong correlation between sprout distances and hydrogel stiffness at day 7, with the longest sprouts emanating from the aorta corresponding to the condition that attained the highest levels of G’. Our lab has recently shown that vascular morphogenesis induces hydrogel stiffening in both fibrin^44^ and PEG^45,46^ hydrogels. Furthermore, microrheological measurements have also revealed local changes in hydrogel G’ values near EC undergoing sprouting angiogenesis^44^, which can establish mechanical gradients that may influence vascular sprouting. Collectively, our findings here suggest that vascular sprouting from macroscale mouse vessels into either acellular or cell-laden fibrin hydrogels shares many similarities to established programs of angiogenic sprouting and vascular assembly observed previously.

Inosculation between the human cell-derived microvasculature and the sprouted mouse EC is also important for the successful creation of a chimeric hierarchical vasculature. We observed interactions between human and mouse EC in hydrogels containing all three macroscale vessel types. Very little is known about the mechanisms by which transplanted microvascular networks inosculate with host vessels upon implantation. A prior study by Cheng, et al. using intravital microscopy in cranial and dorsal window chamber rodent models showed that implanted EC wrapped around nearby host vessels and then induced displacement of the original host endothelium to tap into the existing blood supply. The authors of that study coined the phrase “wrapping and tapping anastomosis” to describe this phenomenon^47^. The morphologies observed in Figs. 4F and 6F here in our study appear remarkably similar, although in our images the mouse EC migrating away from the explanted macrovessels appear to wrap around the human cell-derived microvasculature. We envision this chimeric vasculature could be useful as a model system to further study host-implant inosculation mechanisms and to investigate the influences of age, sex, genetics, and cardiovascular health backgrounds on the formation of vascular anastomoses.

In vertebrate fetal development, formation of the first vessels occurs via vasculogenesis, a process involving angioblast differentiation into either arterial or venous EC followed by self-assembly into immature tubes of either arterial (dorsal aorta) or venous (cardinal vein) origins^48^. These tubes connect to the developing heart and subsequently undergo angiogenic branching, growth, and maturation in response to an increased demand for oxygen delivery and the emergence of convective flow. Capillaries enlarge into arterioles via arteriogenesis, which in turn may enlarge further into larger arteries wrapped with multiple concentric layers of smooth muscle to withstand the hemodynamic forces imposed by blood flow as the need for convective transport arises concurrent with fetal growth. Postnatally, most new blood vessel development occurs via angiogenic sprouting from small caliber vessels. Our findings suggest a complementary phenomenon whereby EC from an undamaged, mature edge of a large caliber macrovessel can sprout either as single cells or clusters into a surrounding acellular and cell-laden hydrogels, respectively. Though technically no longer quiescent following cessation of blood flow through the vessels, this top-down phenomenon suggests a mechanism by which hierarchical vasculature may develop, which in turn may aid efforts to recreate vascular trees for tissue engineering.

While the direct inosculation between sprouts from large vessels and capillaries may not be physiologic per se, prior studies have explored similar approaches to engineer multiscale vasculature. One such study by Chiu, et al. reported the interconnection of two macroscale vessel segments, an artery and a vein, by inducing capillary sprouting from the cut (lumenal) end using topographical and biological cues to guide nascent sprouts^28^. Similarly, another recent study used a 3D printed fenestrated polymeric conduit as a macroscale conduit, and cast cell-laden hydrogel capable of microvascular assembly around this conduit via 3D bioprinting^11^. Endothelial cells seeded within the polymeric conduit were able to integrate and communicate via the fenestrations with the capillary network on the abluminal side, creating a perfusable hierarchical construct capable of being sutured to the rat femoral artery.

Despite the proof-of-concept interconnection of EC across 3 different length scales (Fig. 9), a limitation of our study was the inability to demonstrate perfusion across the hierarchy, suggesting functional inosculation is likely incomplete. The inclusion of mesoscale channels did not significantly alter sprouting morphology in acellular hydrogels but yielded qualitatively more multicellular sprouting in cell-laden hydrogels. These results suggest that our model is amenable to fabrication of an integrated hierarchical vasculature. Our prior work in a lab-on-chip device revealed that integration of meso- and microvasculature formed from EC-LF co-cultures inosculated and perfused 55–75% of the time^30^. While we did not observe perfusion across scales ex vivo in this study, perfusion and subsequent physiologic remodeling in response to hemodynamic forces may occur more rapidly when our constructs are surgically anastomosed in vivo and immediately connected to the blood circulation, enabling a subset of capillaries connected to the macroscale vessel to undergo arteriogenesis. Thus, despite the fact the primitive hierarchy created here is not initially a faithful mimic of native vasculature, this proof-of-concept study lays the groundwork for more rigorous analysis of host-implant inosculation and the engineering of more complex perfusable hierarchical vascular tissue constructs.

## Conclusion

In summary, here we have reported the fabrication of a primitive, multiscale, vascular hierarchy comprising self-assembled microvasculature, micromolded mesovasculature, and explanted murine macrovessels. All 3 large explanted murine vessels evaluated showed evidence of angiogenic sprouting and interactions with human cell-derived microvascular networks when embedded in cell-laden fibrin gels, with the explanted murine vessels of arterial origins exhibiting higher angiogenic potential and capacity to form chimeric vasculature in 3D fibrin gels than those of venous origins. While we were unable to demonstrate functional perfusion across length scales, this work represents a proof-of-concept step towards the creation of constructs containing hierarchical vasculature for applications in regenerative medicine. The more immediate utility is as an experimental model system to provide insight to the process of vascular integration across scales.

## Materials and methods

### Cell culture

Normal human lung fibroblasts (LF; Lonza, Walkersville, MD) were cultured in Dulbecco’s modified Eagle’s medium (DMEM; Gibco, Waltham, MA) supplemented with 10% fetal bovine serum (FBS; Gibco). LF were used between passage 10 and 15. Human umbilical vein endothelial cells (EC) were isolated from fresh umbilical cords obtained from Mott’s Children’s Hospital via an IRB exempt protocol and cultured in endothelial growth medium (EGM-2; Lonza). EC were used between passage 4 and 7. Cell culture media for both cell types were changed every other day. All cells were maintained in 5% CO_2_ at 37 °C.

### Murine vessel harvest

All animal procedures were compliant with the NIH Guide for Care and Use of Laboratory Animals and approved by the University of Michigan’s Institutional Animal Care and Use Committee. The animal study and procedures were consistent with the ARRIVE guidelines. Male BALB/c mice (Taconic Farms, Rensselaer, NY) between 5 and 7 weeks of age were used for this study. Animals were acclimated to their surroundings for 3 days following delivery. Animals were anesthetized using 2% inhaled isoflurane. Fur was removed from the lower legs and abdomen on the ventral side of the animal via shaving followed by the application of depilatory cream to remove residual fur. First the femoral bundles from each leg were removed. An incision was made over the vessels and the fascia was separated from the vessel-nerve bundle. Gentle dissection was used to separate the bundle from the surrounding muscle and subsequently the artery-vein (AV) bundle from the nerve (Fig. 1D). The same procedure was repeated on the second limb. The animal was then euthanized by 5% overdose of isoflurane. Subsequently, the abdominal and thoracic cavities were opened to induce a pneumothorax and to expose the inferior vena cava (IVC) and aorta (Fig. 1C). Adventitia was removed from the IVC and aorta prior to isolation. Vessels were rinsed thrice in sterile saline to remove residual blood and tissue. The aorta was cannulated and flushed with saline through the lumen; other vessels were too small in diameter to do the same.

### Hydrogel fabrication and culture

Individual vessels or vessel bundles were embedded in fibrin hydrogels (cell-laden or acellular) (Fig. 1A). The final composition of resultant hydrogels was 5 mg/mL bovine fibrinogen (Sigma), 1 U/mL bovine thrombin (Sigma), and 10% FBS. Cell-laden hydrogels contained a 1:1 ratio of EC and LF at a final density of 500 K cells/mL. Constructs were cultured to day 1, 7, or 14 in standard EGM-2 or EGM-2 containing 2.2 µM aprotinin (Sigma, St. Louis, MO) with media exchanged on the first day and every other day thereafter.

### Rheological characterization

Hydrogel stiffness was measured using parallel plate shear rheometry on an AR-G2 rheometer (TA Instruments; New Castle, DE) with an 8 mm diameter measurement head. The mechanical properties of hydrogels containing explanted vessels and controls (no explant) were measured by applying a 6% strain amplitude at 1 rad/sec frequency on days 1 and 7 to obtain shear moduli values (G’). Measurements were performed in the culture plates on a stage heated to 37 °C to ensure measurement conditions matched culture conditions as much as possible. Three independent replicates (N = 3) were measured for each condition and time point.

### Immunofluorescent staining and imaging

Constructs were fixed on the last day of culture with Zinc Formalin Fixative (Z-fix; Anatech; Battle Creek, MI) for 15 min at room temperature and washed 3 times for 5 min each with tris buffered saline (TBS). Fixed gels were stained with 4’, 6-diamidino-2-phenylindol (DAPI; 1 µg/L; Fisher Scientific), AlexaFluor 488 Phalloidin (1:200 dilution; Fisher Scientific), DyLight 649-conjugated Griffonia Simplicifolia Lectin I Isolectin B4 (1:50 dilution; Vector Labs, Burlingame, CA), and rhodamine-conjugated Ulex europaeus Agglutinin I (cell-laden only; UEA; 1:200 dilution; Vector Labs). Staining dilutions were made in TBS and samples were incubated with staining solution overnight at 4 °C. Following staining, samples were washed with TBS for at least 24 h.

Samples were imaged on an Olympus IX81 outfitted with a confocal disc spinning unit (DSU; Olympus America, Center Valley, PA) and Metamorph software (Molecular Devices, Sunnyvale, CA). Confocal z-stacks (300 µm, 7 slices) were obtained at 4 × magnification and compressed into maximum intensity projections preceding sprout distance analysis. Additionally, phase contrast, whole-well, single z-plane scans were obtained for analysis of degradation and sprout percentage.

### Analysis of degradation

Acellular samples were imaged in phase using the scan slide application in Metamorph to obtain an entire well image of each construct consisting of an explanted vessel in fibrin. Degradation was measured as the absence of fibrin surrounding the vessel (Fig. 1E). The entire explant perimeter and the degraded perimeter were measured. To obtain a percent degraded metric, the perimeter of the explant that exhibited degradation was divided by the total explant perimeter. Degradation analysis was performed on 3 independent replicates at day 7 only.

### Analysis of sprouting

Multiple metrics were used to assess sprouting from vessel explants into the surrounding fibrin hydrogel. The first metric was a percentage of the explant that exhibited sprouting (Fig. 1F). The perimeter of the explant that exhibited sprouting was divided by the total explant perimeter to obtain a percent sprouted metric. Second, sprout distance was measured as the average distance sprouts reach from the explant edge and was measured only in sprouted regions (Fig. 1G). For this metric, 15+ measurements were taken per explant across multiple images. For all measurements, N = 3 independent replicates were analyzed per condition per timepoint. Lumenal sprouting refers to sprouts originating from the transverse (cut) section of the vessel at the lumen opening. Abluminal sprouting refers to sprouts originating from the vessel wall along the longitudinal edge of the vessel. In the cases where a vessel curled over itself during embedding, analysis was conducted on the external edge only to not skew sprout distance quantification (e.g., in Fig. 4A, femoral the central region between the two ends was not used for distance quantification).

### Fabricating three-scale vascular hierarchies

To fabricate three-scale hierarchies with embedded mesovessels, we fabricated a polydimethylsiloxane (PDMS; Sylgard 184, Ellsworth Adhesives, Germantown, WI) mold with reservoirs to seed arteriole-scale channels with EC (Fig. 1H), as described previously^30^. Briefly, 290 μm acupuncture needles were coated in 2% gelatin (Sigma) and inserted into the PDMS chamber. Cell-laden or acellular hydrogels were fabricated as described above and cast around the needles with an aorta placed in between the two needles. Needles were removed leaving behind empty cavities that were seeded with EC to form mesovessels.

### Statistics

Data were analyzed using Prism 9 (GraphPad, San Diego, CA). All data are represented as mean ± standard deviation. All data points represent a vessel from an individual animal, where, for some types of analyses, multiple measurements were averaged to obtain the data point from each animal. Analysis was conducted on N = 3 explanted vessels from 3 different animals per measurement type. Data were analyzed using a one-way ANOVA with Sidak’s multiple comparisons post hoc test or a nonparametric test with Dunn’s post hoc test. Comparisons were made between vessel types at the same timepoint or between timepoints for the same vessel type. *p* < 0.05 was considered statistically significant.

## Data Availability

The datasets generated analyzed during the current study are available from the corresponding author on reasonable request.
